# Antibacterial Activity of Brass against Antibiotic-Resistant Bacteria following Repeated Exposure to Hydrogen Peroxide/Peracetic Acid and Quaternary Ammonium Compounds

**DOI:** 10.3390/microorganisms12071393

**Published:** 2024-07-09

**Authors:** Emilie Dauvergne, Corinne Lacquemant, Catherine Mullié

**Affiliations:** 1 Laboratoire AGIR—UR UPJV 4294, UFR de Pharmacie, Université de Picardie Jules Verne, 80037 Amiens, France; 2 FAVI SA, 80490 Hallencourt, France; clacquemant@favi.com

**Keywords:** brass, copper, stainless steel, antibacterial activity, peracetic acid, hydrogen peroxide, quaternary ammonium, disinfectant, hospital-acquired infection

## Abstract

Copper-containing materials are attracting attention as self-disinfecting surfaces, suitable for helping healthcare settings in reducing healthcare-associated infections. However, the impact of repeated exposure to disinfectants frequently used in biocleaning protocols on their antibacterial activity remains insufficiently characterized. This study aimed at evaluating the antibacterial efficiency of copper (positive control), a brass alloy (AB+^®^) and stainless steel (negative control) after repeated exposure to a quaternary ammonium compound and/or a mix of peracetic acid/hydrogen peroxide routinely used in healthcare settings. A panel of six antibiotic-resistant strains (clinical isolates) was selected for this assessment. After a short (5 min) exposure time, the copper and brass materials retained significantly better antibacterial efficiencies than stainless steel, regardless of the bacterial strain or disinfectant treatment considered. Moreover, post treatment with both disinfectant products, copper-containing materials still reached similar levels of antibacterial efficiency to those obtained before treatment. Antibiotic resistance mechanisms such as efflux pump overexpression did not impair the antibacterial efficiency of copper-containing materials, nor did the presence of one or several genes related to copper homeostasis/resistance. In light of these results, surfaces made out of copper and brass remain interesting tools in the fight against the dissemination of antibiotic-resistant strains that might cause healthcare-associated infections.

## 1. Introduction

Hospital-acquired infections (HAIs), also known as healthcare-associated infections, have become a major concern for healthcare facilities over the last few decades. This is especially true for HAIs caused by multi-drug-resistant (MDR) pathogens, which are common [1]. Among the preventative measures proposed to mitigate the risk of HAIs, the frequent biocleaning of surfaces to achieve the lowest possible bioburden and avoid the persistence/transmission of antibiotic-resistant pathogenic organisms through this route is recommended [2,3,4]. More recently, the use of self-sanitizing surfaces such as wood or copper alloys has also been proposed as a complementary measure to limit the bioburden on surfaces and, consequently, pathogen transmission induced [5,6].

The antimicrobial effect of copper and derived alloys such as brass has long been acknowledged through different sources, be they historical empirical reports or more recent scientific works [6,7,8,9]. It has even lead the US Environmental Protection Agency (EPA) to reference a wide range of copper alloys containing a minimum amount of 58% copper as antimicrobial surfaces in 2008 [10]. The biocidal effect of copper and copper alloys is also referred to as contact killing. In this mechanism, the metallic surface releases cuprous and cupric ions that act on bacteria, viruses and/or fungi, causing damage to various cellular targets, such as membranes, proteins and nucleic acids [6,8]. The amount of copper contained in copper alloys is a crucial point for their antimicrobial efficiency but is not the only contributing factor. Actually, several other parameters affect the antimicrobial effect, such as alloy components other than copper, surface tension, environmental conditions (hygrometry and temperature), exposure time, inoculum composition and/or deposition volume [11,12,13]. Multiple studies have worked on these parameters to demonstrate the usefulness of brass and other copper alloys in limiting surface contamination in healthcare settings as well as in reducing HAIs [6]. However, just like every other surface, these self-sanitizing surfaces made out of copper alloys can be subjected to biocleaning protocols recommended in infection prevention and control (IPC) measures. As the antimicrobial effect of copper materials depends on the release of ions from their surface, questions have been raised regarding the interference of the repeated use of biocleaning agents on said surfaces and on their antibacterial efficacy. Disinfecting chemical compounds used in IPC protocols for biocleaning but also for the terminal decontamination of rooms can be composed of various chemicals, such as alcohols, glutaraldehyde, quaternary ammonium compounds (QAs), chlorinated derivatives, peracetic acid (PA) and/or hydrogen peroxide (HP) [2]. Few studies have so far reported on their impact on the antimicrobial activity of brass and other copper alloys. The earlier ones report on the effect of a limited number of disinfectants (sodium hypochlorite and ethanol) applied one to five times on surfaces, which does not reflect the possible wear caused to the surface material by the frequent and long-term use of these chemicals, as is the case in healthcare settings [14,15,16]. More recent studies have focused on the residual antibacterial activity of several copper materials following a longer/more frequent application of commercially available disinfectants, such as a 0.5% HP solution, an 8% QA solution or a 5% sodium hypochlorite solution [17,18]. Although these studies gave a better understanding of the durability of copper materials and their antibacterial activity, they did not explore the combined use of disinfectants on self-sanitizing surfaces, which is likely to occur in real life. Moreover, in all these studies, the antibacterial efficacy was assessed on bacterial strains from culture collections, which are not antibiotic-resistant and/or MDR microorganisms.

The aim of this work was therefore to assess the impact of both individual and combined uses of two commercially available products used in French healthcare settings for biocleaning purposes (a QA product and a mix of PA/HP) on a copper alloy. A one-year frequency of their use in hospital was simulated (which will henceforth be called aging process) and the residual antibacterial efficacy was assessed on clinical antibiotic-resistant strains to provide a more realistic estimation. To assess a possible role in survival on copper-containing surfaces, these strains were also genetically characterized for the presence of some of the main determinants of copper homeostasis and/or resistance in bacteria [19]. 

## 2. Materials and Methods

Each experiment was performed at least three times on a minimum of three samples.

### 2.1. Metal Specimens

Three types of metals were used in this study: 304 L stainless steel (negative control for antimicrobial activity)(Thyssenkrupp, Maurepas, France), AB+^®^ brass (62.5% of copper) (FAVI SA, Hallencourt, France) and copper (>90%) (positive control for antimicrobial activity) (FAVI SA). Each metal sample measured 18.05 mm × 19.93 mm. Brass samples were produced using a die-casting foundry process while copper and stainless-steel samples were obtained from sheets by laser cutting. All samples underwent the same surface treatment prior to the aging process using disinfectants.

### 2.2. Aging Process with Disinfectants Routinely Used in Healthcare Settings

To simulate a long-term exposure to frequently used disinfectants and its potential detrimental effect on the antibacterial efficacy of copper and brass, metal specimens were subjected to the repeated application of (i) a QA derivative (Surfa’safe premium^®^, Laboratoires Anios, Lezennes, France), (ii) a mix of PA and HP (Aseptanios AD^®^, Laboratoires Anios) or (iii) a combination of both treatments (QA followed by the PA/HP mix).

The QA derivative (Didecyldimethylammonium chloride, CAS No. 7173-51) was sprayed on non-woven wipes (WypAll^®^, Kimberly-Clark, Nanterre, France), applied on both sides of the metal samples and left to act for 5 min according to the supplier’s recommendations for an optimal antibacterial efficacy [20]. This treatment was applied 365 times to simulate a one-year daily use of the product on surfaces, as is frequently the case in healthcare settings. Similarly, the mix consisting of PA (CAS No. 79-21-0) and HP was selected to simulate a no-touch automated room disinfection (NTD). Placed in airtight plastic boxes (volume 11 L), metal samples were exposed to an atmosphere saturated with the mix for 90 min, longer than the exposure time recommended by the manufacturer for all bacteria but *Bacillus subtilis* spores [21]. As this method is used in healthcare settings for the disinfection of surfaces less frequently than wiping with QA compounds, it was only applied 30 times to simulate a two-to-three times a month frequency over a year. As mentioned above, a combination of exposures to both products was also implemented.

Finally, all samples were subjected to a final cleaning step with acetone in an ultrasound waterbath at 230 V–50 Hz (USC300 T ultrasound waterbath, VWR, Fontenay-sous-Bois, France) for 5 min and rinsed with distilled water. Then, samples were disinfected with 70° ethanol, rinsed once more with sterile distilled water and set to dry under a class 2 biosafety cabinet in sterile Petri dishes prior to seeding with bacterial strains for the antibacterial efficacy assay.

### 2.3. Bacterial Strains

A selection of six clinical and healthcare environmental strains representative of the main bacterial species encountered in HAIs and of various antibiotic resistance mechanisms were drafted (Table 1). This panel included strains displaying an overexpression of efflux pumps, a mechanism known for its versatile role in resistance to antibiotics as well as other antimicrobial molecules, such as biocides and copper [11,22]. Strains were kept at −20°C on cryobeads (VWR, France) until use.

### 2.4. Detection of Genes Involved in Bacterial Copper Homeostasis and Resistance

The six strains included in the panel were grown for 18 to 24 h in Luria Bertani broth (VWR Chemicals, Solon, OH, USA) at 37 °C. Genomic and plasmidic DNAs were extracted from these cultures using the GeneJET Genomic DNA and GeneJET Plasmid Miniprep kits (Thermo Scientific, Takkebijsters, The Netherlands) according to the manufacturer’s instructions. The amount of DNA in each extract was measured using a NanoVue Plus^TM^ spectrophotometer (Biochrom, Thermo Fisher Scientific Inc., Illkirch, France). DNA extracts were amplified using the DreamTaq PCR Master Mix (Thermo Scientific) using primers described in Table S1. Each reaction consisted of 12.5 µL DreamTaq mix, 5 µL of forward and reverse primers at 5 pmol/µL, 5 µL of DNA extract and 5 µL of PCR grade water. Amplifications were carried out in a Veriti^TM^ thermal cycler (Applied Biossytems, Les Ullis, France) and consisted of 1 denaturation cycle at 95 °C for 5 min followed by 35 amplification cycles (30 s at 95 °C followed by 30 s at hybridization temperature and 1 min at 72 °C for elongation) and a final elongation cycle at 72 °C for 10 min. Amplified products were run on a 1.5% agarose gel (Eurogentec, Seraing, Belgium) containing a fluorescent SYBR^®^ Safe stain (VWR, France) and visualized using an IBright 1500 reader (Thermo Fisher, Bourgoin-Jallieu, France).

### 2.5. Antimicrobial Efficacy Testing

The testing was carried out using a previously described worst-case scenario (WCS) method [13] derived from ISO guidelines [23]. Briefly, inocula were prepared with a strain subcultured twice for 24 h and adjusted to MacFarland 4 in sterile saline. An organic soil load was prepared with 30 g/L bovine serum albumin (Merck, Fontenay-sous-Bois, France) and Triton X-100 (Merck, France) at 0.01%. This organic soil load was added to the inocula (6%, *v*/*v*) to mimic the organic contamination found in droplets of saliva or on the skin surface, for example. Inocula were then seeded on metal samples using a non-spread deposit of 1 µL. The contact time between the inoculum and the metal sample (exposure time) was kept to a minimum, corresponding to the drying time of the inoculum, which typically occurred within 5 min (henceforth referred to as a 5 min exposure time). The recovery of viable bacteria was undertaken using 10 mL of Letheen broth (VWR, France) and ultrasonication for 5 min. To enumerate residual viable bacteria, decimal dilutions of the Letheen broth were carried out in sterile saline from 10^−1^ to 10^−3^ and 250 µL was spread in duplicate on Tryptic Soy Agar (TSA) (VWR, France). All plates were then incubated for 48 h at 37 °C prior to enumeration. To lower the detection limit, a filtration step of the residual volume of Letheen broth on a 0.45 µm mixed cellulose esters membrane (Merck Millipore, Darmstadt, Germany) was added. The membrane was placed on TSA and similarly incubated for 48 h at 37 °C.

The results of bacterial enumerations are expressed as log colon-forming unit (CFU)/metal sample and calculated using Equation (1).

Log CFU/metallic sample = log_10_(((CFU count × dilution factor)/0.25) × 10) (1)

The reduction in surviving bacteria between stainless steel 304 L (negative control) and the antimicrobial surfaces (brass and copper) was calculated with Equation (2).

Reduction (%) = 100 − ((∑ brass or copper enumerations/∑ stainless steel enumerations) × 100)(2)

Unless otherwise stated, results are reported as mean ± standard deviation (SD).

### 2.6. Statistical Analysis

Differences between enumerations for stainless steel, brass and copper were computed using a Mann–Whitney test for unpaired samples and Wilcoxon and Friedman tests for paired samples. R software version 3.4.2 (https://www.r-project.org (accessed on 21 June 2024)) and the vassarstats online calculator were used for the calculations. A *p*-value < 0.05 was considered as significant.

### 2.7. Data Availability 

The raw data used to prepare this paper can be accessed online through the following link: https://osf.io/8p2ye/?view_only=4dbd8a3e8e7c48bda47a1d0dd0ede7cc (accessed on 3 July 2024).

## 3. Results

### 3.1. Detection of Genes Involved in Copper Homeostasis and Resistance in Antibiotic-Resistant Strains

Positive amplification results were obtained for all genomic extracts using 16SrDNA primers, validating the conditions in which extractions and amplifications took place. The panel strain in which the highest number of copper-related genes was detected was KPNAM2, with four detected genes out of eight (Table 2). On the contrary, EFUMAM2 only harbored the *czcA* gene, which is related to copper homeostasis through its regulation mechanism [24]. Surprisingly, *cueO* was not detected in any of the six panel strains (Table 2).

### 3.2. Visual Aspect of Metal Surfaces Post-Aging Treatment

**Figure 1:**
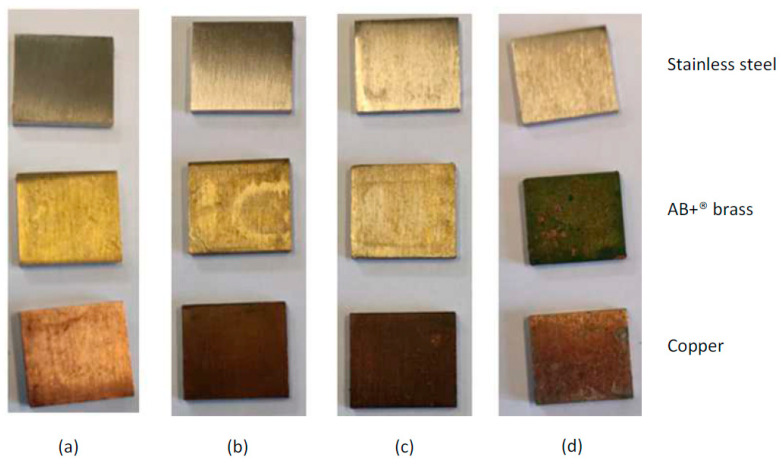
Macroscopic aspect of metal samples: (**a**) untreated; (**b**) post-treatment with a quaternary ammonium compound (365 times); (**c**) post-treatment with a quaternary ammonium compound (365 times) followed by peracetic acid/hydrogen peroxide (30 times); (**d**) post-treatment with peracetic acid/hydrogen peroxide (30 times).

The various aging processes did not induce any visible changes in the stainless steel macroscopic aspect. However, both QA, PA/HP and their combination generated macroscopic variations in the aspect of copper while, for AB+^®^ brass, it only differed following a combined treatment with QA and PA/HP (Figure 1).

### 3.3. Antibacterial Efficacy Post-Aging with a Single Disinfectant

The recovery of each of the antibiotic-resistant strains tested in this study was significantly lower on both copper and AB+^®^ brass untreated surfaces than on the stainless steel one (Table 3). Moreover, the antibacterial efficiency on copper was slightly but significantly better than that on AB+^®^ brass for *Enterobacter cloacae* ECLOAM1 and *Staphylococcus aureus* SAAM33 while AB+^®^ brass was significantly more efficient than copper on *Enterococcus faecium* EFUMAM2 (Table 3). 

The effect of the aging process with QA compounds on the antibacterial activity of metal surfaces was assessed on the whole panel of selected bacteria (Table 3). Both copper and AB+^®^ brass retained a greater antibacterial efficiency than stainless steel post-QA aging process (Table 3). Copper antibacterial activity was slightly but significantly higher than that of AB+^®^ brass for three of the four Gram-negative strains tested but not for Gram-positive strains (Table 3).

The antibacterial efficacy of metal surfaces treated with the sole combination of PA/HP was only evaluated for one Gram-negative and one Gram-positive strain: *Acinetobacter baumannii* ABAM41 and *Staphylococcus aureus* SAAM33. The reduction percentages obtained for these two strains on the copper-positive control were 100 ± 0 and 99.94 ± 0.084, respectively. They were significantly higher than those registered for AB+^®^ brass (97.13 ± 1.021 and 98.85 ± 0.550, respectively) (*p* < 0.001, Mann–Whitney test) but both copper-containing materials still displayed a greater antibacterial efficiency than stainless steel post-treatment with PA/HP (*p* < 0.001, Mann–Whitney test).

### 3.4. Antibacterial Efficacy Post-Aging with a Combination of Quaternary Ammonium Compound and Peraceticacid/Hydrogen Peroxide Mix

The effect of the aging process with the QA and PA/HP combination on the antibacterial activity of metal surfaces was also assessed on the whole panel of selected bacteria (Table 3). Similarly to what was described for both untreated surfaces and QA- or PA/HP-treated ones, the recovery of bacteria on both copper and AB+^®^ was significantly lower than that on stainless steel. As for significant differences in antibacterial efficiency between the two copper-containing surfaces, copper was once more slightly more efficient than AB+^®^ brass on all Gram-negative strains while no significant variations were found for both Gram-positive strains (Table 3).

### 3.5. Comparison of Antibacterial Efficacies following the Different Aging Processes

The trends in antibacterial efficiency following the various aging processes for a single material were also compared, and significant differences were uncovered for all strains on all surfaces, with the single exception of *Enterococcus faecium* EFUMAM2 on copper (Table S2). Pairwise comparisons using a Wilcoxon test highlighted that aging with QA induced a significantly higher recovery of all strains on both stainless steel and AB+^®^ brass as well as those of *Pseudomonas aeruginosa* AM85 and *Klebsiella pneumoniae* KPNAM2 on copper (Tables S2 and S3). However, as mentioned above, bacterial reduction rates remained above 99% for all strains on copper as well as for all strains but *Enterococcus faecium* EFUMAM2 for AB+^®^ brass for QA-treated surfaces. 

Aging with the PA/HP mix also induced a significant increase in the recovery of both strains tested (ABAM41 and SAAM33) on stainless steel and AB+^®^ brass but only that of *Staphylococcus aureus* SAAM 33 on copper (Tables S2 and S3).

Lastly, the combination of QA and PA/HP treatments allowed for recovery rates similar to those of the untreated surfaces for most of the strains on both copper-containing surfaces (Tables S2 and S3). The best reduction rates for AB+^®^ brass were obtained on QA- and PA/HP-treated surfaces, with bacterial reduction rates above 99% for all strains, while only *Klebsiella pneumoniae* KPNAM2 and *Enterococcus faecium* EFUMAM2 failed to reach this mark on QA- and PA/HP-treated copper (Table 3).

It is also noteworthy that, after only 5 min of exposure to the surface and regardless of the copper-related genes detected or the type of antibiotic resistance, bacterial reduction rates remained above 95% in all but two of the forty combinations tested in this work. 

## 4. Discussion

In order to reduce the cross-transmission of potential pathogens to patients through surfaces, iterative disinfection of these surfaces using chemicals is nowadays the standard procedure. However, it represents a time-consuming and fastidious task. It is also challenging for geometrically complex surfaces such as those of beds or wheelchairs, for example. Indeed, notwithstanding the geometrical complexity of the surface, several studies have demonstrated that the majority of surfaces in healthcare rooms are not being properly disinfected [25,26,27]. Alternatives such as copper-containing materials enabling the continuous self-sanitizing of surfaces without the application of cleaning procedures using disinfectants are thus drawing attention. However, even though a no-disinfectant cleaning policy on these self-sanitizing surfaces would present economic (reduced costs in manpower and chemicals) and ecologic (a reduction in environmental and occupational chemical exposure) advantages, it is not yet sufficiently substantiated to be advocated.

Consequently, this work focused on the impact of some widely used disinfectants on such surfaces to evaluate whether they would either decrease or increase their antibacterial activity, either when used alone or in combination. Actually, although reports on the antibacterial efficiency of copper-containing surfaces abound in the scientific literature [6], only a few studies can be found that deal with the impact of disinfectant/cleaning products on this feature [15,16,17,18]. And, to our knowledge, no work has yet been published on the combined use of these products on surfaces, as might be the case in real life. 

The antimicrobial effect of copper is thought to rely on copper ions released from the surface through oxidation [28]. Disinfectants applied on copper-containing materials might either decrease or increase the liberation of copper ions from the treated surface depending on their mode of action. We chose to explore QA and PA/HP disinfectants because they have dissimilar modes of action and so might differently impact the antibacterial efficiency of copper-containing surfaces. Oxidizing agents such as PA or HP would increase the release of copper ions depending on the copper content of the surface [29] while QA compounds would be less likely to do so because they act by destabilizing biological membranes [30]. With regard to brass alloys, dezincification is the most common sort of corrosion encountered, generating modifications in the layers of CuO, Cu_2_O, ZnO and Zn_2_O on brass surfaces [31]. This process could be amplified by combined treatment with QA and PA/HP disinfectants and explain the macroscopic changes in the appearance of the copper alloy under these conditions. The changes in oxides layers could in turn modify the availability of Cu^+^ and Cu^2+^ released from the AB+^®^ brass surface. However, despite the slightly altered macroscopic aspect in AB+^®^ brass, no major differences in antibacterial efficacy were demonstrated regardless of whether or not samples were treated with QA and/or PA/HP.

Indeed, our results show that a maintained antibacterial efficacy, in accordance with the 99% reduction standard issued by the ISO [23], was obtained for all and five out of the six antibiotic-resistant strains tested for QA-treated copper and AB+^®^ brass, respectively. The same level of efficacy was retained following the combined aging process (QA followed by PA/HP) for four strains out of six on copper and all strains on AB+^®^ brass. However, treatment with the PA/HP combination alone resulted in an antimicrobial efficacy on the two tested strains (ABAM41 and SAAM33) below the 99% threshold for AB+^®^ brass, while the copper surface easily reached this cut-off. As for the 99.9% antibacterial efficacy cut-off chosen by the EPA antimicrobial stewardship [10], it was retained for four strains on copper and one strain on AB+^®^ brass post-QA aging. Meanwhile, post QA and PA/HP aging, this value was achieved for three strains on each of the copper-containing surfaces. However, it should be kept in mind that the duration of exposure to the copper-containing surface in this work was only 5 min when the 99.9% value was set based on a 2 h exposure time, which might explain the relative lack of performance witnessed in our work against this cut-off.

A previous study used bleach (a chlorinated derivative), activated HP or a QA compound on surfaces made of (i) integral copper (solid Cu-Ni alloy), (ii) spray-on copper coating—a chemical-free solid metal alloy coating of 80% Cu–20% Ni applied onto hospital-grade stainless steel—and (iii) a Cu-impregnated surface (CIS) [17]. The antimicrobial activity of the solid cupro-nickel alloy against a *P. aeruginosa* and a *S. aureus* strain was the least impacted by the presence of all disinfectants, which led us to work on solid brass rather than other types of brass to ensure a better durability. This previous study also pointed out that HP slightly reduced the antibacterial efficiency of integral copper on *P. aeruginosa* while QA appeared to synergize with released copper ions from non-integral copper [17]. This synergy between copper ions and QA compounds was also hypothesized in another study on *P. aeruginosa* biofilms [32]. A further study concluded that no significant modification to the antistaphylococcal and antipseudomonal efficiency of copper-containing materials was induced by a prolonged (200 times) treatment with either QA or accelerated HP [18], which is more in accordance with the results obtained here on a different type of integral alloy.

To better simulate what would happen in real healthcare settings/hospital life, we not only chose to use commercially available disinfectants for this study but also clinical antibiotic-resistant strains. Most if not all studies on the antibacterial efficiency of copper-containing materials previously mentioned making use of collection strains, recommended in standardized protocols. However, these strains do not display antibiotic resistance features, which are quite common in strains found in the environment of healthcare settings. It must also be underlined that some of the antibiotic resistance mechanisms found in antibiotic-resistant bacteria have been linked to a cross-resistance with copper and/or other metals. Efflux pump (EP) systems are one example. They have been described as participating in copper homeostasis and resistance in *P. aeruginosa* [33]. This is why the ciprofloxacin-resistant *P. aeruginosa* AM85 strain, previously described as overexpressing several resistance–nodulation–division EPs [34], was chosen in this study. The gene encoding another EP, *copA*, was also detected here in this strain, along with *czcA*. This latter gene encodes an EP more specific to Zn^2+^ but has been shown to be regulated by a system that can be activated by copper [35]. Moreover, it has been linked with carbapenem resistance [24,35]. Vancomycin-resistant enterococci (VRE) are another example of the association of metal and antibiotic resistances with the concomitant presence of glycopeptide resistance genes and *tcrB* encoding an ATPase efflux pump [36]. However, this concomitant presence was not witnessed in the VRE strain chosen in our panel (EFUMAM2). Despite the various mechanisms of antibiotic resistance and copper-related genes harbored by the selected strains, AB+^®^ brass and copper retained a good antibacterial efficiency, regardless of the disinfectant treatment applied. This is an encouraging point for advising the use of such copper-containing materials in healthcare settings. In addition, from an aesthetic point of view, AB+^®^ brass might be more easily accepted by hospital staff and patients, as its macroscopic aspect is less impacted than that of pure copper by the repeated applications of disinfectants. Also, from an economic point of view, surfaces made of copper alloys would be less costly to implement than surfaces made of pure copper (as estimated from reclaimed metal costs in June 2024 for pure copper and brass: USD 3.75 and USD 2.25, respectively). 

Some limitations found in this work should nevertheless be taken into account before drawing definitive conclusions. The first is that we only focused on a couple of disinfectants and their combination. It might be interesting to expand this work using other widely used disinfectants, such as alcohols, chlorinated derivatives and their combinations. This would help in broadening the assumption that solid integral copper-containing surfaces such as brass can withstand the chemical and mechanical wear induced by IPC biocleaning protocols and retain their antibacterial properties. Concerning mechanical wear, we made the choice of applying the commercially available disinfectants as performed in real life, i.e., by hand for QA and aerosolization for PA/HP. We did not use a crockmeter or another specially designed apparatus, which was proposed in previous studies [15,16,17,18] and would have allowed for a standardized repetitive application of QA wipes, for example. Moreover, apart from the macroscopic aspect of metal samples, this work did not focus on the analysis of the surfaces using techniques such as scanning electron microscopy (SEM), Fourier transform infrared spectroscopy (FTIR) or X-ray photoelectron spectroscopy (XPS) before and after aging. It would indeed be interesting in future endeavors to try and link changes in surfaces post-aging with the retained antibacterial efficacy. Finally, to better define relationships between the determinants in antibiotic and copper resistances, it might also have been interesting to select a panel of strains first for their ability to resist copper and afterward to see if they displayed antibiotic resistance mechanisms and not the other way around, as was carried out in this study.

## 5. Conclusions

Copper-containing materials retained a good antibacterial efficiency post-aging with either a QA disinfectant or a combination of QA and PA/HP disinfectants against a panel of both Gram-negative and Gram-positive antibiotic-resistant bacteria. The recovery of bacteria after a short (5 min) exposure time to solid copper and AB+^®^ brass was consistently lower than on stainless steel before and after aging with disinfectants. The presence of various mechanisms of antibiotic resistance and genes related to copper homeostasis/resistance did not markedly impact the antibacterial efficiency of the tested copper-containing materials.

## Figures and Tables

**Table 1:** Characteristics of the clinical strains included in the antimicrobial efficacy testing.

Strain Reference	Bacterial Species	Antibiotic Resistance Profile ^1^	Sample Origin (Isolation Year)
ABAM41	*Acinetobacter baumannii*	Oxa-23, AmpC, ArmA	Environment (2017)
AM85	*Pseudomonas aeruginosa*	EPO	Rectal swab (2009)
ECLOAM1	*Enterobacter cloacae*	Oxa-48, ESBL	External quality control (2019)
EFUMAM2	*Enterococcus faecium*	*vanA*	Sputum (2008)
KPNAM2	*Klebsiella pneumoniae*	KPC	Rectal swab (2017)
SAAM33	*Staphylococcus aureus*	*mecA*, EPO	Tracheal (2012)

^1^ AmpC: cephalosporinase; ArmA: aminoglycoside resistance methylase; EPO: efflux pump overexpression; ESBL: extended-spectrum beta-lactamase; KPC: *Klebsiella pneumoniae* carbapenemase; *mecA*: gene encoding the penicillin-binding protein 2A, displaying a reduced affinity for β-lactams; Oxa-23 and Oxa-48: carbapenemases; *vanA*: gene cluster conferring resistance to glycopeptides.

**Table 2:** Genes detected in the clinical strains included in the antimicrobial efficacy testing.

Gene	Function	ABAM41	AM85	ECLOAM1	KPNAM2	EFUMAM2	SAAM33
*copA*	ATPase pump	−/+ ^a^	+/+	−/−	−/−	−/−	−/−
*tcrB*	ATPase pump	+/+	−/−	−/−	−/−	−/−	+/−
*cusA*	RND ^1^ pump	−/−	−/−	−/−	+/+	−/−	−/−
*pcoD*	Inner membrane pump	−/−	−/−	+/+	+/+	−/−	−/−
*czcA*	Zn^2+^ pump	+/+	+/+	−/−	+/+	+/−	+/−
*cueO*	Multicopper oxidase	−/−	−/−	−/−	−/−	−/−	−/−
*pcoE*	Chaperone	−/−	−/−	+/+	+/+	−/−	−/−
*copZ*	Chaperone	−/−	−/−	−/−	−/−	−/−	+/−

^1^: Resistance–nodulation–division efflux pump; ^a^: genomic/plasmidic detection (−: negative; +: positive).

**Table 3:** Antibacterial efficacy of copper and AB+^®^ brass expressed as reduction percentages compared to stainless steel before (untreated) and after aging with a quaternary ammonium compound (QA) or a combination of a quaternary ammonium compound with a mix of peracetic acid and hydrogen peroxide (QA and PA/HP) (*n* = 3, exposure time = 5 min).

Bacterial Strain	Copper			AB+^®^ Brass		
	Untreated	QA	QA and PA/HP	Untreated	QA	QA and PA/HP
ABAM 41	93.15 ± 11.517 ^1,^*	99.99 ± 0.019 *	100 ± 0 *	99.95 ± 0.051 *	99.27 ± 0.420 *^$^	99.91 ± 0.137 *^†^
AM85	99.95 ± 0.068 *	99.99 ± 0.008 *	100 ± 0 *	100 ± 0 *	99.86 ± 0.087 *	99.99 ± 0.017 *^†^
ECLOAM1	99.73 ± 0.342 *	99.93 ± 0.059 *	100 ± 0 *	99.44 ± 0.913 *	99.63 ± 0.072 *^$^	99.99 ± 0.001 *^†^
KPNAM2	98.03 ± 2.343 *	99.54 ± 0.200 *	96.75 ± 5.622 *	99.16 ± 0.582 *	99.20 ± 0.255 *^$^	99.77 ± 0.398 *^†^
EFUMAM2	76.15 ± 27.228 **	99.03 ± 0.587 *	96.45 ± 3.133 *	99.94 ± 0.050 *	97.96 ± 0.046 *	99.38 ± 0.936 *
SAAM33	99.97 ± 0.053 *^,£^	100 ± 0 *	99.89 ± 0.175 *	99.85 ± 0.129 *^,£^	100 ± 0 *	99.81 ± 0.090 *

^1^ Results expressed as mean ± standard deviation. Significant difference in the numbers of recovered colony-forming units on the negative control surface (stainless steel) and on the other metallic surface (copper or AB+^®^ brass) (Mann–Whitney test) at *: *p* < 0.001 and **: *p* < 0.01. ^£^: Significant difference in the numbers of recovered colony-forming units on untreated copper and AB+^®^ brass (*p* < 0.05, Mann–Whitney test). ^$^: Significant difference in the reductions on copper and on AB+^®^ brass surfaces post-QA treatment (*p* < 0.001, Mann–Whitney test). ^†^: Significant difference in the reductions on copper and on AB+^®^ brass post-QA and PA/HP treatment (*p* < 0.001, Mann–Whitney test).

## Data Availability

The original data presented in the study are openly available in the Open Science Framework (OSF) repository at https://osf.io/8p2ye/?view_only=4dbd8a3e8e7c48bda47a1d0dd0ede7cc (accessed on 3 July 2024).

## Supplementary Materials

The following supporting information can be downloaded at: https://www.mdpi.com/article/10.3390/microorganisms12071393/s1, Table S1: Genes involved in bacterial copper homeostasis and/or resistance and primers used for their amplification; Table S2: Colony-forming units recovered on untreated and disinfectant-treated metallic surfaces (QA: quaternary ammonium treatment; PA/HP: peracetic acid/hydrogen peroxide treatment; QA and PA/HP = quaternary ammonium combined with peracetic acid/hydrogen peroxide treatment); Table S3: *p*-values of pairwise comparisons calculated by Wilcoxon test (QA: quaternary ammonium treatment; PA/HP: peracetic acid/hydrogen peroxide treatment; QA and PA/HP = quaternary ammonium combined with peracetic acid/hydrogen peroxide treatment). References [37,38,39,40,41,42,43,44,45] are cited in the Supplementary Materials.
