# Supplementary material for: Antibacterial Activity of Brass against Antibiotic-Resistant Bacteria following Repeated Exposure to Hydrogen Peroxide/Peracetic Acid and Quaternary Ammonium Compounds
Source: Microorganisms. 2024 Jul 9;12(7):1393. doi: 10.3390/microorganisms12071393 (PMC11279221; doi:10.3390/microorganisms12071393)
Supplement: Supplementary file 1 [file microorganisms-12-01393-s001.zip › microorganisms-3093183-supplementary.pdf]

Table S1. Genes involved in bacterial copper homeostasis and/or resistance and primers used for their amplification.

Target gene	Function		5'→3' sequence	Hybridization (°C)	Amplicon size (bp)	Reference
tcrB	ATPase pump	FWD	CATCACGGTAGCTTTAAGGAGATTTTC	55	663	[37,38]
		REV	ATAGAGGACTCCGCCACCATTG			
pcoD (*pcoABCDRS*)	Inner membrane pump	FWD	CTGGCCACACTTGCCTGGGG	55	500	[37,38]
		REV	CACGCTACGGCGCCCAGAAT			
16S rDNA	Ribosomal RNA subunit	FWD	ACTCCTACGGGAGGCAG	55	196	[39]
		REV	ATTACCGCGGCTGCTGGCA			
copA (*copABCDRS*)	ATPase pump	FWD	ATGTGGAACSARATGCGKATGA	61	193	[40]
		REV	AGYTTCAGGCCSGGAATACG			
cusA (*cusCFBA*)	RND pump	FWD	ATGCSACVGGYGTTGGCTGG	61	410	[41]
		REV	CCRTTCAGYTCGGCRATRCC			
czcA (*czcAB*)	Zn^{2+} pump	FWD	TCGACGGBGCCGTGGTSMTBGTCGAGAA	61	232	[40]
		REV	GTVAWSGCCAKCGGVBGGAACA			
cueO	Multicopper oxidase	FWD	CCCTAGGCGGTGTTTTCATA	48	996	[42,43]
		REV	TCATTGTCAAGGCAACCAA			
pcoE	Chaperone	FWD	ATGAATATATTAATCACGAC	48°C	450	[44]
		REV	TTACCTGGTCGAATACAGCC			
copZ (*copYZAB*)	Chaperone	FWD	AAAATTGATGGGATGAAATG	48°C	179	[45]
		REV	AACCTTGAATTTTGTATCTGC			

Table S2. Colony forming units recovered on untreated and disinfectant-treated metallic surfaces (QA: quaternary ammonnium treatment; PA/HP: peracetic acid/hydrogen peroxide treatment; QA & PA/HP = quaternary ammonium combined with peracetic acid/hydrogen peroxide treatment).

Bacterial strain	**Stainless steel**				**Copper**				**AB+® brass**			
	**Untreated**	**QA**	**PA/HP**	**QA & PA/HP**	**Untreated**	**QA**	**PA/HP**	**QA & PA/HP**	**Untreated**	**QA**	**PA/HP**	**QA & PA/HP**
**ABAM41**[*,**]												
Median	240000	582000	520000	624000	80	1	0	0	5	2200	4600	1
Minimum	20000	156000	80000	128000	0	0	0	0	0	50	80	0
Maximum	432000	1360000	1080000	1032000	28400	400	4	21	800	30000	22800	3600
**AM85**[*,**]												
Median	25800	98000		16200	0	2		0	0	78		0
Minimum	0	36000	ND[1]	80	0	0	ND	0	0	0	ND	0
Maximum	180000	252000		58800	3	17		0	0	2400		23
**ECLOAM1**[*,**]												
Median	71000	520000		820000	44	46		0	0	1200		1
Minimum	4000	200000	ND	208000	2	3	ND	0	0	4	ND	0
Maximum	216000	1040000		4400000	800	2400		1	2000	8000		47
**KPNAM2**[*,**]												
Median	3800	266000		186000	8	400		0	3	1200		0
Minimum	80	92000	ND	56000	0	13	ND	0	0	80	ND	0
Maximum	28400	1000000		532000	400	7600		132000	800	12400		2800
**EFUMAM2**[*]												
Median	400	142000		188000	46	208		1	0	800		3
Minimum	0	53600	ND	28800	0	0	ND	0	0	1	ND	0
Maximum	10000	272000		348000	8800	4400		26000	800	19600		9600
**SAAM33**[*,**]												
Median	76000	224000	860000	520000	0	0	30	4	46	0	5800	80
Minimum	11600	30000	2000	52000	0	0	2	0	0	0	800	0
Maximum	1560000	876000	1920000	1480000	800	1	2800	10400	1200	15	31600	2800

[1]: Not determined

Significant difference between the various conditions [*]: for stainless steel and AB+® brass ($p<0.001$, Friedman test) and [**]: for copper ($p<0.05$, Friedman test)

Table S3. p-values of pairwise comparisons calculated by Wilcoxon test (QA: quaternary ammonnium treatment; PA/HP: peracetic acid/hydrogen peroxide treatment; QA & PA/HP = quaternary ammonium combined with peracetic acid/hydrogen peroxide treatment).

	**Stainless steel**			**Copper**			**AB+® brass**		
	**QA**	**PA/HP**	**QA & PA/HP**	**QA**	**PA/HP**	**QA & PA/HP**	**QA**	**PA/HP**	**QA & PA/HP**
**ABAM41**									
**Untreated**	**0.0003**[*]	**0.0022**	**0.0003**	0.0536	**0.0024**	**0.0045**	**<0.0001**	**<0.0001**	0.8966
QA		0.2713	0.6892		**0.0128**	**0.0366**		**0.0434**	**<0.0001**
**PA/HP**			0.5287			>0.05			**<0.0001**
**AM85**									
**Untreated**	**0.008**	ND[1]	0.234	**0.0024**	ND	>0.05	**<0.0001**	ND	0.05
QA		ND	**0.0003**		ND	**0.0024**		ND	**<0.0001**
**PA/HP**			ND			ND			ND
**ECLOAM1**									
**Untreated**	**0.0002**	ND	**0.0002**	0.2543	ND	**0.0002**	**<0.0001**	ND	**0.0155**
QA		ND	**0.0193**		ND	**0.0002**		ND	**<0.0001**
**PA/HP**			ND			ND			ND
**KPNAM2**									
**Untreated**	**0.0002**	ND	**0.0002**	**0.0034**	ND	0.1118	**<0.0001**	ND	0.5552
QA		ND	0.5619		ND	**0.0285**		ND	**0.0008**
**PA/HP**			ND			ND			ND
**EFUMAM2**									
**Untreated**	**0.0002**	ND	**0.0002**		NS[2]		**0.002**	ND	0.0561
QA		ND	0.1615					ND	**0.0025**
**PA/HP**			ND						ND
**SAAM33**									
**Untreated**	**0.0226**	**0.0111**	**0.0036**	0.05	**0.0324**	0.6312	**<0.0001**	**<0.0001**	0.0601
QA		**0.0117**	**0.0088**		**<0.0001**	**0.003**		**<0.0001**	**<0.0001**
**PA/HP**			0.1236			0.7188			**<0.0001**

[*]: Values in bold reflect a statistically significant difference between the two compared conditions

[1]: Not determined; [2]: Not significant (p>0.05, Friedman test)
